# The Hidden Diverticula: A Case Report on Appendiceal Diverticulosis

**DOI:** 10.7759/cureus.65813

**Published:** 2024-07-31

**Authors:** Murad M Hamiedah, Moath R Alzboon, Hussien R Al-Nawaiseh, Omar H Makhamreh, Yasmeen K Alsoboh

**Affiliations:** 1 General Surgery, Prince Ali Bin Al-Hussein Military Hospital, Jordanian Royal Medical Services, Amman, JOR; 2 Histopathology, Princess Iman Center for Research and Laboratory, Jordanian Royal Medical Services, Amman, JOR

**Keywords:** appendicular neoplasia, diverticulosis of the appendix, appendicular diverticulosis, appendicitis, appendiceal diverticulosis

## Abstract

Diverticulosis of the appendix (DA) is a rare condition, often asymptomatic and incidentally discovered, with a significant association with neoplasia. The prevalence of neoplasia in specimens without DA versus those with DA was 1.28% and 26.94%, respectively. Here, we discuss a case of a 54-year-old male presented with left flank pain and dysuria. Examination showed left renal angle tenderness and leukocytosis. CT scan revealed a left ureteric stone and an enlarged appendix. The urology team placed a double-J catheter, and surgical consultation led to an appendectomy. Histopathology confirmed DA with acute inflammation, serositis, and fibroblast proliferation without malignancy. This case underscores the need to consider DA in differential diagnoses and the importance of thorough histopathological examination and timely surgical intervention.

## Introduction

Diverticulosis of the appendix (DA) is a relatively rare pathological finding, with a reported prevalence ranging from 0.014% to 3.7% [1,2]. Most cases are acquired pseudodiverticula, while true congenital diverticula are exceedingly rare. Typically, appendiceal diverticulosis is an incidental finding and clinically asymptomatic. A systematic review and meta-analysis revealed a significant association between DA and neoplasia [2]. Here, we present a case of appendiceal diverticulosis, incidentally discovered and confirmed through histopathology.

## Case presentation

**Figure 1:**
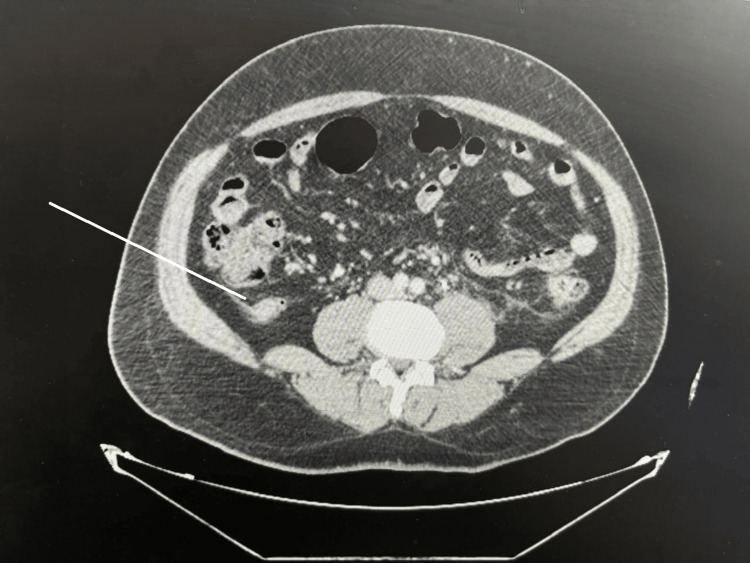
Abdomen CT scan image The image shows the 1.4 cm dilated appendix with fat stranding.

A 54-year-old male with a 20-year history of renal stones presented to the emergency department with a complaint of left flank pain and dysuria of one day's duration. The patient did not experience anorexia or right lower quadrant pain. Upon examination, he was afebrile with stable vital signs. Physical examination revealed left renal angle tenderness without right lower quadrant tenderness or rebound tenderness. Laboratory results showed a white cell count of 13.5 × 10^3^/µL(Reference range: 4.5-10 × 10^3^/µL). A CT scan demonstrated a 5 mm left ureteric stone, causing hydronephrosis and perinephric fat stranding. Additionally, the appendix measured 1.4 cm in diameter, surrounded by periappendiceal fat stranding and lymphadenopathy, suggestive of chronic inflammatory changes, though malignancy could not be excluded (Figure 1).

**Figure 2:**
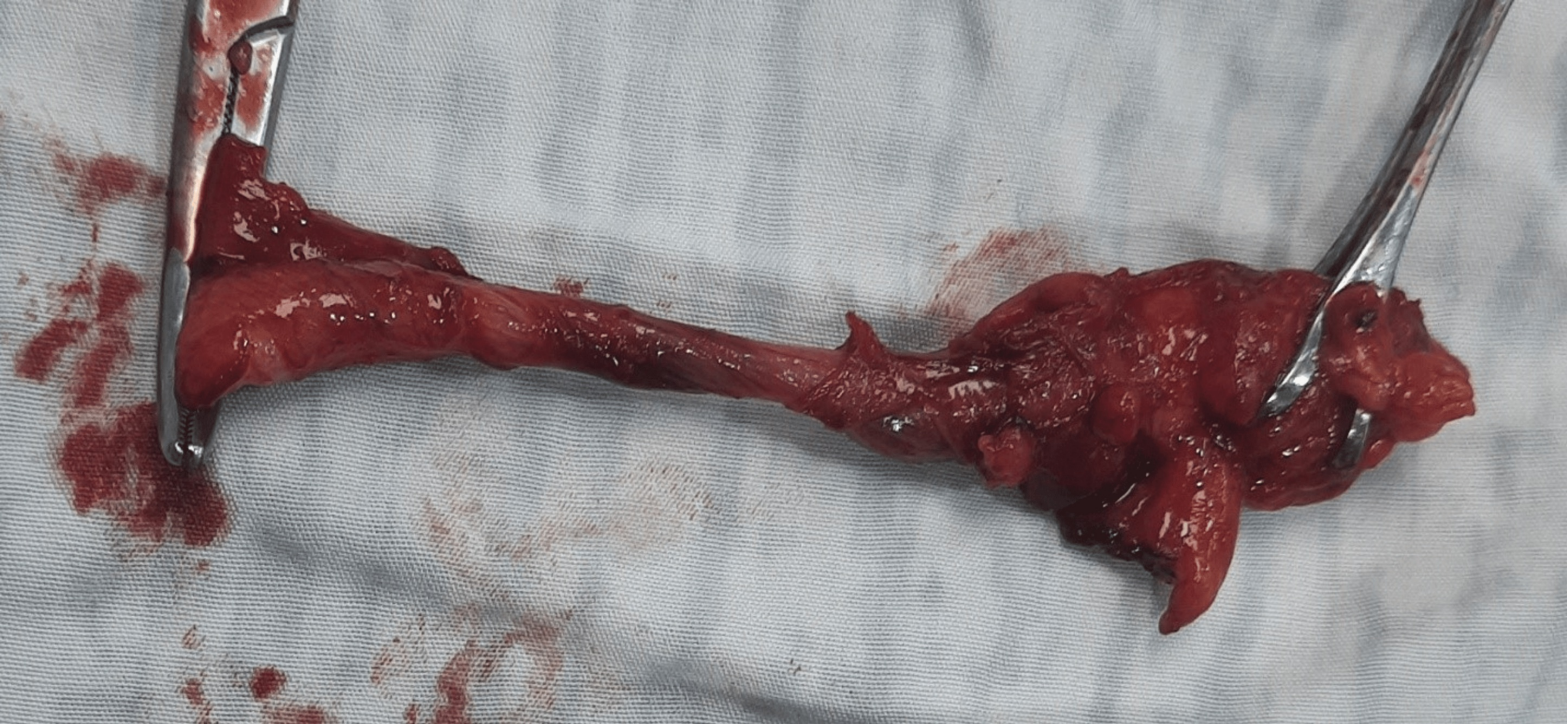
Gross appendix specimen showing diverticulosis

The urology team was consulted, and a double-J catheter was placed to manage the kidney stones. Given the patient's asymptomatic status regarding the appendix, a surgical consensus led to the decision for an appendectomy. Diagnostic laparoscopy was initiated but converted to an open procedure due to difficult dissection and chronic peritoneal adhesions, along with the suspicion of a mass. Gross examination revealed an enlarged appendiceal tip (>2 cm) with a healthy cecum (Figure 2). An appendectomy was performed, with a plan to await histopathology results before considering a right hemicolectomy. The patient was discharged without postoperative complications.

**Figure 3:**
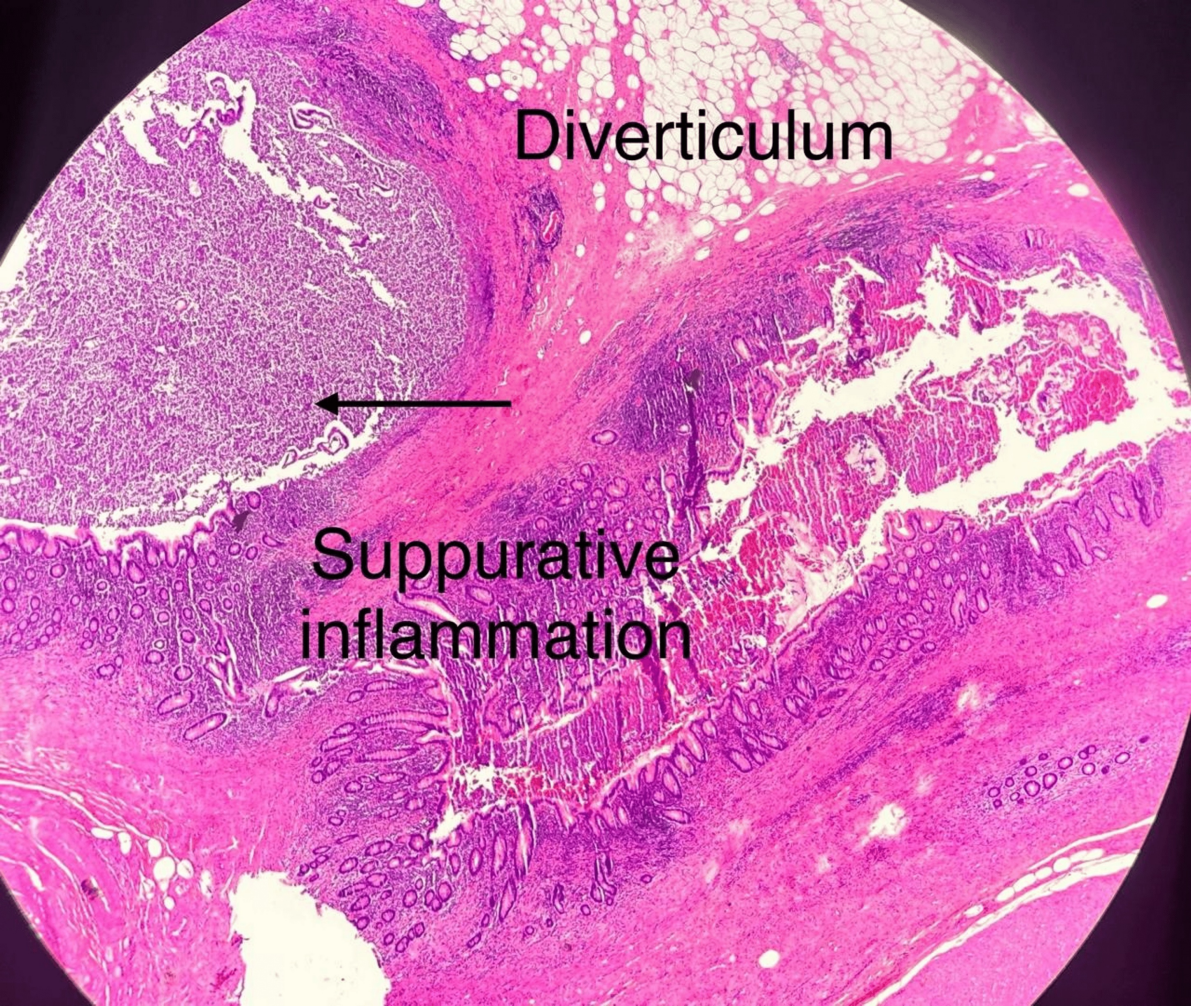
Histopathology section showing diverticulum with suppurative inflammation

**Figure 4:**
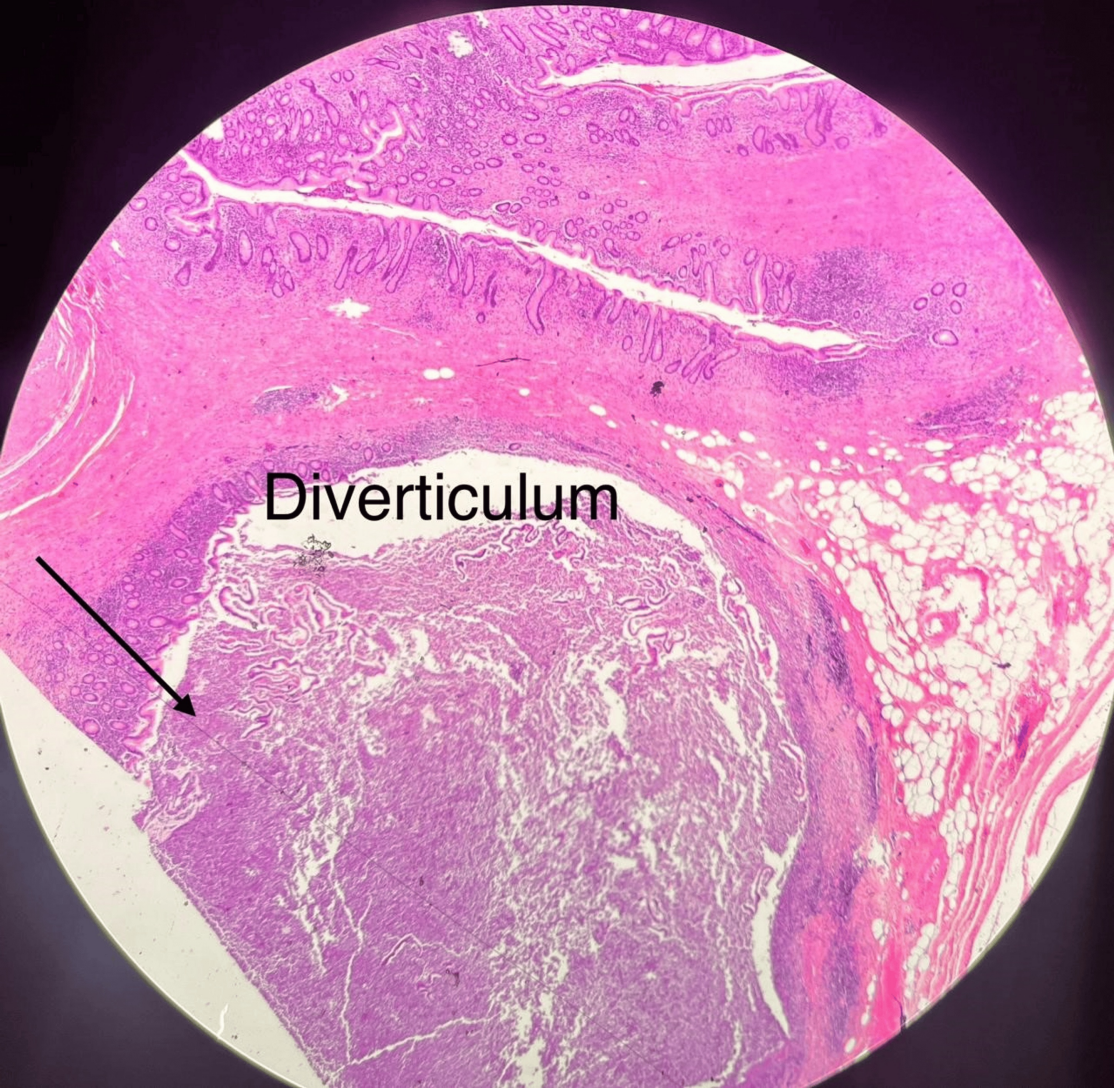
Histopathology section showing diverticulum in the appendix

Histopathology showed dilation at the appendiceal tip, covered with exudate, pus, and mucus. The final diagnosis was appendiceal diverticulosis associated with acute on chronic suppurative inflammation, serositis, and fibroblast proliferation, with no evidence of malignancy (Figures 3, 4).

## Discussion

Several case reports and series highlight the clinical presentations, management, and outcomes of appendiceal diverticulosis. A case series published in the American Journal of Case Reports discussed various presentations of DA, emphasizing its rarity and the importance of distinguishing it from acute appendicitis. The series also underscored the potential complications, including perforation and association with neoplasms [3,4].

In another case report, acute perforated appendicitis was linked to appendiceal diverticulitis in a young man, highlighting the condition's potential severity and the need for prompt surgical intervention [5]. Similarly, a literature review found that appendiceal diverticulitis could present with acute gastrointestinal bleeding, requiring combined diagnostic approaches for accurate preoperative identification [6].

Two types of DA have been identified: congenital and acquired. The acquired type, which is the most prevalent, is a false diverticulum, representing a herniation of the mucosa through a muscular defect of the appendix, primarily on the mesenteric border. The exact pathogenesis remains unclear, but several theories have been proposed. The inflammatory theory suggests that an episode of appendicitis results in wall weakness, leading to ulceration and subsequent epithelial regeneration over the injured area. Stout proposed that luminal obstruction and muscular contraction cause high intraluminal pressure, forming a diverticulum at the mesenteric border where the artery enters [7,8]. Other theories suggest a multifactorial origin.

DA has been further classified into four subtypes: Type 1 (acutely inflamed diverticulum with a normal-appearing appendix), Type 2 (acutely inflamed diverticulum with surrounding appendicitis), Type 3 (conventional appendicitis with incidental non-inflamed diverticulum), and Type 4 (incidental diverticulum without appendicitis or diverticulitis) [4].

Treatment involves surgical removal due to the high risk of perforation and associated morbidity and mortality. Unlike classical left-sided diverticulitis, appendiceal diverticulitis requires elective appendectomy. Laparoscopic treatment is not contraindicated. A meta-analysis examining the association between DA and neoplasia revealed a significant correlation, suggesting that patients with DA are at higher risk for appendiceal neoplasms compared to the general population [2,9]. Given the high association between appendiceal diverticula and neoplasia, careful macroscopic and microscopic examination is essential, as appendiceal diverticula may play a role in the pathogenesis of appendiceal mucinous tumors [3,5,10].

## Conclusions

Appendiceal diverticular disease is infrequent and easily overlooked. Given its strong association with appendiceal neoplasms, thorough examination of the appendix post-appendectomy and careful follow-up of histopathology reports are crucial. Considering the association with malignant conditions, urgent appendectomy for appendiceal diverticulitis and elective appendectomy for incidental findings of appendiceal diverticulosis should be recommended.
